# EGFR-TKI不良反应管理专家共识

**DOI:** 10.3779/j.issn.1009-3419.2019.02.01

**Published:** 2019-02-20

**Authors:** 中国抗癌协会肺癌专业委员会

**Affiliations:** 200030 上海，上海交通大学附属上海市胸科医院肿瘤科 Department of Oncology, Shanghai Chest Hospital Afliated to Shanghai Jiaotong University, Shanghai 200030, China

**Keywords:** EGFR-TKI, 肺肿瘤, 不良反应, 专家共识, EGFR-TKI, Lung neoplasms, Adverse drug reaction, Consensus

## Abstract

表皮生长因子受体酪氨酸激酶抑制剂（epidermal growth factor receptor-tyrosine kinase inhibitors, EGFR-TKI）如吉非替尼、厄洛替尼、埃克替尼和阿法替尼在中国已成为*EGFR*突变阳性的晚期非小细胞肺癌（non-small cell lung cancer, NSCLC）的一线治疗药物，奥希替尼也获批用于二线治疗*EGFR*-T790M突变阳性的晚期NSCLC。EGFR-TKI的常见不良反应（adverse drug reaction, ADR）有皮疹、腹泻、甲沟炎、口腔粘膜炎、肝损伤、间质性肺疾病等。中国抗癌协会肺癌专业委员会根据中国国内不良反应诊疗现状，结合国际最新理论和经验，组织相关专家讨论并制定了EGFR-TKI不良反应管理中国专家共识。

## Abstract (en)

ErbB receptor tyrosine kinase inhibitors (EGFR-TKI), gefitinib, erlotinib, icotinib and aftinib, which are approved as a frontline treatment for patients with non-small cell lung cancer (NSCLC) who have tumors harboring *EGFR* mutations in China. And osimertinib was approved in second line setting for patients with EGFRT 790M-positive NSCLC. Rash, paronychia, diarrhea, stomatitis, liver dysfunction and (interstitial lung disease, ILD) are frequently observed in patients treated with EGFR-TKI. Chinese Society of Lung Cancer, Chinese Anti-Cancer Association, organized Chinese experts to develop the Chinese expert consensus on EGFR-TKI adverse event (AE) management based on domestic diagnosis and treatment of ADR and also incorporating international updated theory and recommendations.

针对表皮生长因子受体（epidermal growth factor receptor, *EGFR*）突变阳性晚期非小细胞肺癌（non-small cell lung cancer, NSCLC）一线治疗，多个随机对照研究^[1-11]^显示，吉非替尼、厄洛替尼、埃克替尼和阿法替尼对比化疗均可显著改善患者的无进展生存时间（progression free survival, PFS），且3级及以上不良反应显著低于化疗，奠定了吉非替尼、埃克替尼、厄洛替尼和阿法替尼在*EGFR*突变晚期NSCLC一线治疗的地位。四个药物均获国家食品药品监督管理总局（China Food and Drug Administration, CFDA）批准用于一线治疗*EGFR*敏感突变的晚期NSCLC。此外，对于一代、二代*EGFR*-TKI治疗耐药且经检测有*EGFR* T790M突变阳性的晚期NSCLC患者，在Mok^[12]^研究中，对比标准化疗，奥希替尼可显著延长该类患者的PFS（10.1个月 *vs* 4.4个月，*P* < 0.001）。奥希替尼于2017年3月22日被中国CFDA批准用于EGFR-TKI治疗时或治疗后出现疾病进展、并经检测确认存在*EGFR* T790M突变阳性的局部晚期或转移性NSCLC成人患者的治疗。

相比化疗，EGFR-TKI有其特有的不良反应，如包括皮疹、腹泻、甲沟炎、口腔粘膜炎、肝损伤、间质性肺疾病（interstitial lung disease, ILD）等。目前我国临床上对EGFR-TKI所致的不良反应尚无统一的诊治策略和防治措施，因此，如何管理好EGFR-TKI导致的不良反应，提高患者接受EGFR-TKI药物治疗的依从性，成为一个紧迫的问题。

为此中国抗癌协会肺癌专业委员会牵头，邀请国内消化科、口腔科、皮肤科、呼吸科、中医科及肿瘤科等多位专家共同拟定本共识，针对国内已经获批上市的EGFR-TKI药物导致不良反应的发生率、高危因素、诊断和分级标准以及防治措施进行讨论及总结，旨在为临床医生提供切实可行的诊疗措施，延长晚期NSCLC患者的生存，提高生活质量，使患者治疗获益最大化。

本共识中的证据等级及推荐等级标准见表 1和表 2。

**1:** 证据等级^[13, 14]^ Levels of evidence^[13, 14]^

级别	定义
1	基于至少一项大型随机、对照、良好质控的临床研究，或基于质量控制及一致性良好的随机研究的荟萃分析
2	基于小型随机临床研究，或存在偏倚风险的大型随机研究，或对这类研究以及存在异质性临床研究的荟萃分析
3	基于前瞻性队列研究
4	基于回顾性队列研究或病例对照研究
5	基于未设对照组的临床研究、病例报道或专家意见

**2:** 推荐级别^[13, 14]^ Grades of recommendation^[13, 14]^

级别	定义
A	强有力的疗效证据，具有确定的临床获益，强烈推荐
B	强或中等的疗效证据，但临床获益有限，一般推荐
C	疗效证据不足或获益并不大于风险、缺点（不良事件、成本等），可选
D	中等证据表明疗效不佳或不良结局，一般不推荐
E	强有力的证据表明疗效不佳或不良结局，不推荐

## 1 EGFR-TKI相关性消化系统不良反应及其处理

EGFR-TKI导致的最常见的消化系统不良反应主要包括腹泻和肝损伤。因此，在EGFR-TKI治疗前应告知患者及其家属治疗中可能出现的腹泻和肝损伤风险，并配合医生在治疗过程中进行严密监测，以期早期诊断，并采用恰当的治疗方案和策略。

### 1.1 EGFR-TKI相关性腹泻及其处理

#### 1.1.1 EGFR-TKI相关性腹泻的发生率

EGFR-TKI导致腹泻的确切机制尚不明确，有研究^[5]^提示可能与氯离子的过度分泌有关。EGFR-TKI治疗后，腹泻发生的频率较高，甚至可能出现临床上较为严重的腹泻。在目前已公布的不同EGFR-TKI的Ⅲ期临床研究中，腹泻的总体发生率报道为9.5%-95.2%，≥3级的发生率为1%-14.4%^[1, 2, 5, 6, 8-11]^（表 3）。

**3:** 不同EGFR-TKI的相关Ⅲ期临床研究中腹泻发生率 Incidence of diarrhoea in phase Ⅲ clinical trials about different EGFR-TKI

EGFR-TKI	研究缩写	区域	腹泻	
			所有%	≥3级%
吉非替尼	IPASS^[1]^	东亚	46.6	3.8
	NEJ 002^[2]^	日本	34.2	0.9
厄洛替尼	EURTAC^[5]^	欧洲	57.0	5.0
	OPTIMAL^[6]^	中国	25.0	1.0
阿法替尼	LUX-Lung 3^[8]^	全球	95.2	14.4
	LUX-Lung 6^[9]^	亚洲	88.3	5.4
奥希替尼	AURA 3^[11]^	全球	41.0	1.0
埃克替尼	CONVINCE^[10]^	中国	9.5	7.4
EGFR-TKI：表皮生长因子受体酪氨酸激酶抑制剂				

#### 1.1.2 EGFR-TKI相关性腹泻的临床诊断

腹泻的临床表现主要为大便次数明显增多和大便性状的改变^[16]^。通常，腹泻时的大便性状可表现为稀便、水样便、粘脓便或脓血便。严重腹泻时，患者可出现口渴、皮肤粘膜弹性变差等脱水症状，少数患者还会伴有明显中毒症状（烦躁、精神萎靡、嗜睡、面色苍白、高热或体温不升、外周白细胞计数明显增高等）表现^[16]^。

对于EGFR-TKI治疗前无腹泻而治疗后出现腹泻症状者，或EGFR-TKI治疗前已有腹泻而治疗后腹泻症状显著加重者，均应考虑EGFR-TKI导致腹泻的可能性。因此，其诊断依据应包括明确的服用EGFR-TKI史，以及出现与服药有时间关联的腹泻相关临床症状。

肿瘤患者通常伴随肠道菌群紊乱^[17]^，而肠道菌群紊乱可导致腹泻^[18]^。而且，肿瘤患者免疫功能低下，是肠道感染的高风险人群^[19]^，一旦出现肠道病原学微生物感染，也可出现腹泻症状^[19]^。此外，一些肿瘤如神经内分泌肿瘤的类癌综合征、胃泌素瘤、血管活性肠肽（vasoactive intestinal peptide, VIP）瘤等，疾病本身也可导致腹泻的发生^[20, 21]^。因此，在建立EGFR-TKI所致腹泻诊断时，应同时排除或鉴别其他原因导致的腹泻。由于会影响到后续EGFR-TKI的治疗策略，排除其他原因导致的腹泻是至关重要的。

#### 1.1.3 EGFR-TKI相关性腹泻的临床评估

临床上，一旦诊断为EGFR-TKI所致腹泻，应对腹泻进行合理评估，了解腹泻的严重程度，为后续治疗决策提供依据。

目前，通用的分级标准是美国国立癌症研究所（National Cancer Institute, NCI）针对消化道不良反应制订的分级标准。2017年NCI发布了更新的常见不良反应术语评定标准（common terminology criteria for adverse events, CTCAE）5.0标准分级。腹泻采用的是CTCAE 5.0分级标准^[22]^（表 4）。

**4:** 腹泻的分级标准^[22]^ Grades criteria for diarrhea^[22]^

分级	描述
1	与基线相比，大便次数增加每天 < 4次；与基线相比，造瘘口排出物轻度增加
2	与基线相比，大便次数增加每天4次-6次；与基线相比，造瘘口排出物中度增加；日常生活中工具使用受限
3	与基线相比，大便次数增加每天≥7次；需要院治疗；与基线相比，造瘘口排出物重度增加；日常生活中自理能力受限
4	危及生命；需要紧急治疗
5	死亡

除按上述标准进行分级外，尚应对下列内容进行评估^[23]^：（1）确认出现腹泻症状的时间及持续时间；（2）记录排便次数及排便性状；（3）评估是否有发烧、晕眩、痉挛等症状，以排除伴随其他更严重副作用的可能；（4）评估患者的饮食特点与对先前治疗的依从性。

#### 1.1.4 EGFR-TKI相关性腹泻的预防

对于EGFR-TKI所致腹泻的预防措施主要包括以下内容^[24]^：获得患者接受EGFR-TKI治疗开始前6周的大便信息，以便更好评估EGFR-TKI导致腹泻的状况；在治疗开始前收集患者同时服用的其他药物以及其他临床状况，以便评估药物对消化系统潜在的影响，对可能导致消化系统不良反应的药物相互作用也应进行评估；EGFR-TKI治疗期间应低脂低纤维饮食，忌食用咖啡因、酒精、奶制品、脂肪、纤维、橘子汁、葡萄汁以及辛辣食物，少食多餐；不得服用泻药，除非有医嘱。

#### 1.1.5 EGFR-TKI相关性腹泻的处理

对EGFR-TKI导致腹泻的患者，处理措施可参考表 5。

**5:** EGFR-TKI相关性腹泻的处理措施^[24]^ Management of diarrhoea in patients treated with EGFR-TKI^[24]^

分级	管理	治疗
1级-2级	（1）密切观察，避免脱水；停用软便剂，每天饮用1升等渗液体； （2）改变饮食（避免摄取乳制品、清淡饮食、少量多餐）； （3）第2级腹泻持续时间超过48 h：评估是否有脱水或电解质失衡的状况，并考虑给予输液，每天饮用1 L-1.5 L等渗液体。	（1）使用相同剂量的EGFR-TKI继续治疗；（2）使用洛哌丁胺（5A）、益生菌和思密达（5B）。洛哌丁胺从4 mg开始（2片），在此之后，每次腹泻后、或每隔4 h服用2 mg（1片）（最高剂量16 mg/d），直到排便停止达12 h为止； （3）第2级腹泻持续时间超过48 h将EGFR-TKI暂停用药，并继续使用洛哌丁胺（最高剂量16 mg/d）（5A）、益生菌和思密达（5B）治疗，加用可待因（30 mg *Bid*）（5A）直到缓解至第1级以下，降低EGFR-TKI原剂量，以低剂量重启治疗
3级以上	（1）让患者住院监测，并采集粪便样本进行显微镜检查； （2）每天饮用1 L-1.5 L等渗液体积极给予静脉输液补充至少24 h	（1）暂停使用EGFR-TKI直到缓解至1级及以下，降低EGFR-TKI原剂量，以低剂量重启治疗； （2）使用洛哌丁胺（最高剂量16 mg/d）（5A）； （3）益生菌和思密达（5B）继续治疗，加用可待因（30 mg *Bid*）（5A）；若患者的嗜中性粒细胞增加，则考虑给予预防性抗生素治疗： ①严重时，可考虑加用生长抑素 ②治疗后腹泻于14 d内没有缓解至1级及以下，应给予最佳支持疗法并将EGFR-TKI停用
腹泻物含有大量有害细菌，会导致皮肤损伤、疼痛，可用温水均匀地清洁肛门附近区域，去除有害细菌^[24]^		

#### 1.1.6 EGFR-TKI相关性腹泻的中医论治

##### 1.1.6.1 病因病机

EGFR-TKI相关性腹泻属中医“泄泻”的范畴。其发生责之于体虚和药毒二因，因虚则泻，因泻愈虚，其本质是以虚为主的虚实夹杂证。病位以脾为主，与肝、肾二脏密切相关。

《景岳全书·泄泻》曰：“泄泻之本，无不由于胃”。“肾为胃之关，开窍于二阴，所以二便之开闭，皆肾脏之所主。今肾中之阳气不足，则命门火衰，而阴寒极盛之时，则令人洞泄不止也”。“凡遇怒气便作泄泻者，必先以怒时夹食，致伤脾胃。”此类泄泻，或因久病而脾胃虚弱，或因情志抑郁而肝郁乘脾，或因日久伤肾，肾阳虚而脾失温煦，最终脾胃运化失常、内生湿滞，且易感寒湿，而致泄泻，此为体虚内因。EGFR-TKI是攻伐之品，属中医“药毒”的范畴，易伤脾胃而致泄泻，此为药毒病因。

##### 1.1.6.2 辨病辨证

###### 1.1.6.2.1 辨轻重缓急

泄泻急者发病快，病程较短；泄泻缓者发病缓慢，病程较长。泄泻轻者，泄泻次数少，量少，兼证轻（按分级1级-2级）；泄泻重者，泄泻次数多，量多，兼证重（≥3级）。

###### 1.1.6.2.2 辨寒热

大便色黄而臭秽，肛门灼热者，多属热证；大便清稀，或完谷不化者，多属寒证。

###### 1.1.6.2.3 辨虚实

急性暴泻，腹痛甚而拒按，泻后痛减，多属实证。若有恶寒，发热，头身疼痛，鼻塞，脉浮，多兼表证；若有肢体困重，大便粘腻不爽，苔腻脉濡，多兼湿证；若情志抑郁，胸胁胀痛，多兼气郁。

慢性久泻，反复发作，腹痛不甚，喜温喜按，神疲乏力，多属虚证。若有神疲乏力，大便时溏时烂，则脾虚明显；形寒肢冷，腰膝酸软，大便溏稀，完谷不化，多发生于五更，则肾阳虚明显。

##### 1.1.6.3 辨证分型

根据中医理论及临床经验，其证型主要可分为以下三种。

###### 1.1.6.3.1 脾胃虚弱证

证候：大便时溏时泻，绵延反复，食少，腹胀，进食油腻食物则大便次数增加，伴面色萎黄，神疲倦怠，舌淡，苔白，脉细弱。

###### 1.1.6.3.2 肝气乘脾证

证候：泄泻肠鸣，矢气频繁，嗳气食少，因情志抑郁或精神紧张而发，舌淡红，脉弦。

###### 1.1.6.3.3 肾阳虚衰证

证候：大便溏稀，完谷不化，腹部喜温，泻后则安，多发生于黎明前，伴形寒肢冷，腰膝酸软，舌淡苔白，脉沉细。

##### 1.1.6.4 治则治法

EGFR-TKI所致腹泻的病机，本在于脾胃虚弱，标在于湿浊阻滞。因此，治疗上宜以健脾益气以扶其本虚，并以行气化湿以解其标实，并随证加减。

###### 1.1.6.4.1 主方

藿香正气丸加减：

【组成】藿香15 g、大腹皮10 g、白芷10 g、紫苏叶10 g、茯苓10 g、法半夏15 g、白术15 g、陈皮10 g、厚朴10 g、桔梗10 g、甘草6 g。

【用法】水煎至250 mL，分温内服。

【功效】理气和中、化湿止泻。

【主治】外感风寒，内伤湿滞证，见脘腹胀痛，呕吐泄泻，头痛昏重，胸膈痞闷，舌苔白腻，脉浮或濡缓者。

脾虚胃弱者：加用党参25 g、白扁豆25 g、莲子25 g、人参10 g、山药20 g、薏苡仁30 g等以健脾益气；（注：人参阳虚者以红参或高丽参，阴虚者以西洋参，可另炖服用）。

食滞伤胃者：加用山楂15 g、砂仁6 g后下、鸡内金10 g等以消食和胃；

肝气郁结者：加用柴胡10 g、郁金10 g、川楝子10 g等以疏肝解郁；

郁而化热者：加用黄连3-6 g、栀子10 g等以清热燥湿；

肾阳虚衰者：加用肉豆蔻6 g、补骨脂10 g、五味子6 g、吴茱萸3 g、附子10 g、肉桂6 g等以温肾散寒；

久泻脱肛者：加用黄芪30 g、升麻15 g、干姜10 g等以升阳止泻。

###### 1.1.6.4.2 敷脐方

敷脐止泻散：

【组成】干姜、肉桂、补骨脂、白术、制附子、黄连等量。

【用法】打细粉，每次适量，外敷肚脐，以胶布固定。

【功效】温脾补肾、清热化湿。

【主治】各种腹泻。根据具体情况加减，食谷不化者，加莱菔子、山楂等量；偏热者，去制附子，加栀子等量；偏寒者，加吴茱萸；气滞者，加木香、厚朴等。

##### 1.1.6.5 中医各家学说

多数中医医家认为，EGFR-TKI相关性腹泻多虚证，治法以温补脾肾为主，兼顾兼证，必要时适当收涩。温补脾肾又有侧重之分，神疲乏力，纳差，大便时溏时泻，舌淡苔白，脉细弱者，重在温脾。腰膝酸软，畏冷肢寒，大便完谷不化，脉沉细者，重在温肾。泄泻甚者，急则治标，可适当使用诃子、肉豆蔻等收涩药。切忌过早使用收涩药或收涩太过，以免有闭门留寇之虞。情志抑郁、胸胁胀闷不舒者为肝郁之象，重在疏肝健脾。肿瘤患者常症候复杂，临床上应四诊合参，辨证论治，灵活用药。同时，此类患者应注重饮食调护，忌食生冷和油腻的食物。必要时可结合中医外治法（中药敷脐、中药灌肠），针灸等。

近年来，针对靶向药相关性腹泻的中医临床研究很多。孙等^[25]^研究报道，在吉非替尼治疗前后患者存在中医证候变化，对于吉非替尼治疗肺癌后患者，热毒伤阴，余毒未净为其主要病机特点，治疗当以养阴解毒为主，忌重用苦温燥湿。王等^[26]^临床工作中发现，靶向药物在中医角度看来是苦寒伤中的攻伐之品，泄泻多由脾虚有湿，下趋大肠而致，故治以温运脾胃、利湿止泻为主，临床上有良好收效。张某^[27]^研究发现，参苓白术颗粒能明显降低吉非替尼/厄洛替尼治疗中的腹泻发生率[联合组28.6% *vs* 对照组70%（*P* < 0.05），2b级]。贺某等^[28]^报道，中药腹泻方对NSCLC患者靶向治疗引起的轻中度腹泻的治疗效果优于易蒙停[第14天时试验组缓解率81.82% *vs* 对照组76.67%（*P*=0.029），2b级]。翟某^[29]^研究发现，在吉非替尼治疗基础上给予益肺汤治疗肺癌脑转移头痛患者中，治疗组腹泻发生率为较对照组明显降低[治疗组10.0 *vs* 对照组27.08%（*P* < 0.05），2b级]。林某^[30]^用益气养阴化痰解毒中药能减少吉非替尼引起的腹泻发生[治疗组发生率6.7% *vs* 对照组16.7%（*P* > 0.05），2b级]。

中医药治疗腹泻的优势在于缓解腹泻的同时综合调节患者胃肠道功能，增进食欲，以及可预防腹泻的发生。

传统经典方简便廉验，根据临床情况辨证使用中成药，如脾胃虚弱者，可选用参苓白术丸、香砂六君丸、理中丸等；肝气乘脾证，可选用逍遥丸合香砂六君丸；肾阳虚衰者可选四神丸、桂附理中丸等。

### 1.2 EGFR-TKI相关性药物性肝损伤（drug-induced liver injury, DILI）及其处理

#### 1.2.1 EGFR-TKI相关性DILI的发生率

在目前已公布的不同EGFR-TKI的Ⅲ期临床研究中，DILI的发生率为5%-55.3%，≥3级的发生率为0.4%-26.3%^[1, 2, 5, 6, 8-11]^（表 6）。除阿法替尼以外，多数EGFR-TKI主要通过肝脏（cytochrome P450, CYP450）酶系代谢^[31]^。有研究^[32]^认为EGFR-TKI的肝毒性与其活性代谢产物的代谢有关，而诱导的自身免疫性损伤是EGFR-TKI肝毒性的另一种机制^[33]^。

**6:** 不同EGFR-TKI的相关Ⅲ期临床研究中DILI的发生率 Incidence of DILI in phase Ⅲ clinical trials

EGFR-TKI	研究缩写	区域		发生率	
				所有(%)	≥3级(%)
吉非替尼	IPASS^[1]^	东亚	转氨酶升高	文中未提及相关数据	9.4
	NEJ002 ^[2]^	日本	转氨酶升高	55.3	26.3
厄洛替尼	EURTAC^[5]^	欧洲	转氨酶升高	6	2
	OPTIMAL^[6]^	中国	谷丙转氨酶升高	37	4
阿法替尼	LUX-Lung 3^[8]^	全球	转氨酶升高	文中未提及相关数据	文中未提及相关数据
	LUX-Lung 6^[9]^	亚洲	谷丙转氨酶升高谷草转氨酶升高	20.1 15.1	1.7 0.4
奥希替尼	AURA 3^[11]^	全球	谷丙转氨酶升高谷草转氨酶升高	6 5	1 1
埃克替尼	CONVINCE ^[10]^	中国	谷丙转氨酶升高谷草转氨酶升高	9.5 9.5	2.0 2.7
EGFR-TKI：表皮生长因子受体酪氨酸激酶抑制剂					

#### 1.2.2 EGFR-TKI相关性DILI的临床诊断

##### 1.2.2.1 EGFR-TKI相关性DILI的临床表现和实验室检查

DILI的临床表现通常无特异性^[34]^。部分患者可有乏力、食欲减退、厌油、肝区胀痛及上腹不适等消化道症状。淤胆明显者可有全身皮肤黄染、大便颜色变浅和瘙痒等。少数患者可有发热、皮疹、嗜酸性粒细胞增多甚至关节酸痛等过敏表现，还可能伴有其他肝外器官损伤的表现^[34]^。因此，对于EGFR-TKI治疗过程中出现相应非特异性症状的患者，应考虑到DILI的可能性。但无症状患者，并非一定能排除DILI可能。

血清谷丙转氨酶（alanine aminotransferase, ALT）、谷草转氨酶（aspartate transaminase, AST）、碱性磷酸酶（alkaline phosphates, ALP）、谷氨酰转肽酶（glutamyl transpeptidase, GGT）和总胆红素（total bilirubin, TBil）等改变是目前判断是否有肝损伤和诊断DILI的主要实验室指标^[35]^。此外，白蛋白和凝血功能是反映肝脏功能的指标，其水平下降通常提示肝脏损伤较重，肝脏的功能受到了影响^[34]^。EGFR-TKI治疗过程中，定期筛查这些实验室指标有助于早期发现潜在的DILI。超声、计算机断层扫描（computed tomography, CT）或磁共振成像（magnetic resonance imaging, MRI）等常规影像学检查可协助发现特殊类型的DILI（如失代偿期肝硬化、肝脏血管病变、肿瘤等），在有强烈临床提示时，可考虑进行相应检查。事实上，影像学检查也是临床上DILI鉴别诊断过程中常用的手段^[34]^。

##### 1.2.2.2 EGFR-TKI相关性DILI的临床诊断和鉴别诊断

DILI的诊断目前仍属排他性诊断。在实践中，对疑似DILI患者，首先要确认患者是否存在肝损伤，其次需排除引起肝损伤的其他病因，最后通过因果关系评估量表（Roussel Uclaf Causality Assessment Method, RUCAM）（附录1 http://www.lungca.org/files/2019-1-supp1.pdf）来确定肝损伤与可疑药物EGFR-TKI的因果关系。通常，RUCAM量表打分在6分以上时，可建立DILI诊断；3分-5分时是否可建立诊断目前仍存有争议。诊断的具体流程和策略可参考中华医学会药物性肝病学组发布的《药物性肝损伤诊治指南》^[34]^，如图 1。完整的DILI临床诊断应包括诊断命名、临床类型、病程、RUCAM评分结果、严重程度分级。

**1:**
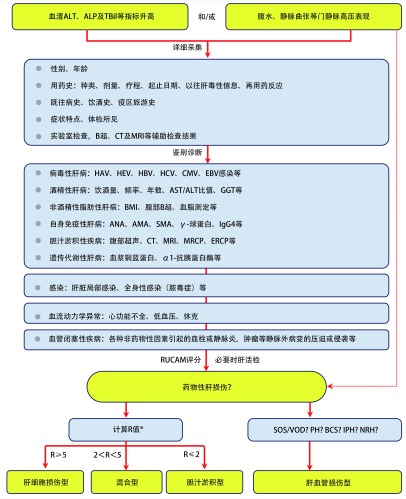
DILI诊断流程图。甲型肝炎病毒（hepatitis avirus, HAV）；戊型肝炎病毒（hepatitis E virus, HEV）；巨细胞病毒（cytomegalovirus, CMV）；EB病毒（Epstein-Barr virus, EBV）；谷氨酰基转移酶（glutamyltransferase, GGT）；身体质量指数（Body Mass Index, BMI）；B型超声（type-B ultrasonic ultrasonic test, B超）；抗核抗体（antinuclear antibody, ANA）；抗线粒体抗体（anti-mitochondrial antibody, AMA）；抗平滑肌抗体（anti-smooth muscle antibody, SMA）；磁共振胰胆管造影（magnetic resonance cholangiopancreatography, MRCP）；内镜下逆行胰胆管造影（endoscopic retrograde cholangiopancreatography, ERCP）；巴德-基亚里综合征（Budd-Chiari syndrome, BCS）；特发性门静脉高压症（idiopathic portal hypertension, IPH）；结节性再生性增生（nodular regenerative hyperplasia of the liver, NRH）；紫癜性肝病（peliosis hepatitis, PH）；肝窦阻塞综合征/肝小静脉闭塞病（sinusoidal obstruction syndrome/veno-occlusivedisease, SOS/VOD）：^*^R＝[ALT实测值/上限（upper limit of normal value, ULN）]/（ALP实测值/ULN）。 Diagnostic algorithm of DILI

由于（1）DILI的潜伏期差异很大，可短至数日至1个月、长达数月，药物和肝损伤的关系比较隐秘；（2）药物可以导致目前已知的所有急性、亚急性和慢性肝损伤类型；（3）同一种药物在不同人群中可导致不同的肝损伤类型；（4）中国有大量慢性乙型肝炎病毒携带者、慢性乙型肝炎和脂肪肝等慢性肝病背景的患者等，这些因素导致了DILI的诊断和鉴别诊断充满挑战。尽管DILI的组织学无特异性表现，但进行鉴别诊断时，尤其是自身免疫性肝炎（autoimmune hepatitis, AIH）不能除外时，肝脏组织学可以提供额外的信息帮助我们进行鉴别诊断。因此，在遇到肝损伤病因诊断仍不明确、疑难和重症肝损伤患者时，建议转诊至肝病专业协助诊治。

需要提醒的是，对肿瘤患者而言，患者除了应用EGFR-TKI外，有可能同时服用其他药物包括其他处方和非处方药、传统中药、保健品等治疗，而这些信息患者可能不会主动告知医生，因此，在建立DILI诊断时，肝损伤是由EGFR-TKI引起的还是服用其他药物引起的，应注意鉴别。

##### 1.2.2.3 DILI的临床分型

诊断DILI后，应对肝损伤进行临床分型。对于EGFR-TKI所致肝损伤，目前无证据显示可造成肝脏血管损伤。因此，对多数患者，分型可参考中华医学会药物性肝病学组发布的《药物性肝损伤诊治指南》^[34]^进行：（1）肝细胞损伤型：ALT≥3×ULN，且R≥5；（2）胆汁淤积型：ALP≥2×ULN，且R≤2；（3）混合型：ALT≥3×ULN，ALP≥2×ULN，且2 < R < 5。R=（ALT实测值/ALT ULN）/（ALP实测值/ALP ULN）。

#### 1.2.3 EGFR-TKI相关性DILI的临床评估

对所有诊断为DILI的患者，可参考中华医学会药物性肝病学组发布的《药物性肝损伤诊治指南》^[34]^，评估肝损伤的严重程度，见表 7。

**7:** 肝损伤的分级标准^[34]^ Grades criteria for liver injury^[34]^

分级	描述
1	轻度肝损伤：血清ALT和/或ALP呈可恢复性升高，TBil < 2.5×ULN (2.5 mg/dL或42.75 *μ*mol/L)，且国际标准化比值（international normalized ratio, INR） < 1.5。多数患者可适应。可有或无乏力、虚弱、恶心、厌食、右上腹痛、黄疸、瘙痒、皮疹或体质量减轻等症状
2	中度肝损伤：血清ALT和(或) ALP升高，TBil≥2.5×ULN，或虽无TBil升高但INR≥1.5。上述症状可有加重
3	重度肝损伤：血清ALT和(或)ALP升高，TBil≥5×ULN (5 mg/dL或85.5 *μ*mol /L)，伴或不伴INR≥1.5。患者症状进一步加重，需要住院治疗，或住院时间延长
4	ALF：血清ALT和（或）ALP水平升高，TBil≥10×ULN（10 mg/dL或171 *μ*mol/L或每天上升≥1.0 mg/dL (17.1 *μ*mol/L)，INR≥2.0或凝血酶原活动度（prothrombin time activity, PTA） < 40%，可同时出现（1）腹水或肝性脑病；或（2）与DILI相关的其他器官功能衰竭
5	致命：因DILI死亡，或需接受肝移植才能存活
ALT：血清谷丙转氨酶；TBil：总胆红素；ALP：碱性磷酸酶；ULN：上限；ALF：急性肝衰竭/亚急性肝衰竭；DILI：药物性肝损伤	

#### 1.2.4 EGFR-TKI相关性DILI的预防

对于EGFR-TKI所致肝损伤的预防主要从以下几个方面进行：认真阅读EGFR-TKI类药物的说明书，了解药物肝毒性的整体情况，了解药物应用的禁忌证和注意事项；用药后严密监测肝损伤的发生，定期进行肝脏生化学检测；遵循说明书和临床指南合理用药；联合应用CYP3A4酶抑制剂或诱导剂，应对EGFR-TKI进行剂量调整；加强用药知情同意管理，提高患者对EGFR-TKI所致肝损伤风险的意识；肝功能改变严重或恶化时，考虑终止用药等。

#### 1.2.5 EGFR-TKI相关性DILI的处理

DILI的基本治疗原则：（1）及时停用可疑肝损伤药物；（2）充分权衡停药引起原发病进展和继续用药导致肝损伤加重的风险；（3）根据肝损伤的临床类型选用适当的药物治疗；（4）急性肝衰竭/亚急性肝衰竭（acute liver failure/subacute liver failure, ALF/SALF）等重症患者必要时可考虑紧急肝移植^[34]^。

由于在多数人群中机体对药物的肝毒性可产生适应性，ALT和AST的暂时性波动临床上很常见，真正进展为严重肝损伤和肝衰竭的情况相对少见，因此，多数情况下血清ALT或AST升高而无症状者并非是必须立即停药的指征。但出现TBil和（或）INR升高等肝脏明显受损的情况时，若继续用药则有诱发ALF/SALF的危险^[34]^，重度或怀疑有急性肝功能衰竭征象者，应及时转诊到专科医生，必要时行肝移植治疗。DILI的具体治疗措施见表 8。

**8:** EGFR-TKI相关性DILI的治疗措施^[34]^ Management of DILI in patients treated with EGFR-TKI^[34]^

治疗措施	具体内容
停药	（1）及时停用可疑的肝损伤药物是最为重要的治疗措施； （2）下述标准是美国FDA在药物临床试验中建议的停药标准，临床上可作为停药的参考。因此，出现下列情况之一建议应考虑停用EGFR-TKI： ①血清ALT或AST > 8×ULN； ②ALT或AST > 5×ULN，持续2周； ③ALT或AST > 3×ULN，且TBil > 2×ULN或INR > 1. 5； ④ALT或AST > 3×ULN，伴逐渐加重的疲劳、恶心、呕吐、右上腹疼痛或压痛、发热、皮疹和（或）嗜酸性粒细胞增多（> 5%）。
药物治疗	（1）重型成人患者可选用N-乙酰半胱氨酸（N-acetyl-L-cysteine, NAC）（2A），临床越早应用效果越好。成人一般用法: 50 mg/kg/d-150 mg/kg/d，总疗程不低于3 d； （2）糖皮质激素应仅限用于超敏或自身免疫征象明显、且停用EGFR-TKI后生化指标改善不明显甚或继续恶化的患者（5B）； （3）异甘草酸镁可用于治疗ALT明显升高的急性肝细胞型或混合型肝损伤（1A）； （4）有经验表明，轻-中度肝细胞损伤型和混合型DILI，炎症较重者可试用双环醇和甘草酸制剂；炎症较轻者可试用水飞蓟素。胆汁淤积型DILI可选用熊去氧胆酸（ursodeoxycholic acid, UDCA）。有报道腺苷蛋氨酸（S-adenosylmethionine, SAMe）治疗胆汁淤积型DILI有效。上述药物的确切疗效有待严格的前瞻性随机对照研究加以证实（5B）。
ALT：血清谷丙转氨酶；AST：谷草转氨酶；INR：国际标准化比值；TBil：总胆红素；ULN：上限；DILI：药物性肝损伤	

关于停药后，能否再用同一个EGFR-TKI或换成其他的EGFR-TKI进行治疗，目前没有明确的共识和推荐。日本曾报道过一例*EGFR*突变阳性肺腺癌的日本老年女性患者，在使用吉非替尼16周后出现肝毒性（AST和ALT升高，毒性级别分别为1级和3级），停药后肝酶恢复至正常水平。换用厄洛替尼治疗10周，肝酶再次升高。停药2周，再次使用厄洛替尼，剂量从100 mg/d减至50 mg/d，直至肝酶恢复正常水平。随后换用阿法替尼，治疗超过44周，未发现肝毒性的证据。作者认为，这是首次使用阿法替尼成功治疗经吉非替尼和厄洛替尼治疗出现高级别肝毒性的案例报道^[35]^。另一项研究^[36]^使用吉非替尼治疗8例NSCLC患者出现AST和ALT升高，随后吉非替尼停用或减量，然后患者均换用厄洛替尼，虽有3例患者出现肝毒性（2例1级、1例2级），但均能耐受，因此，作者认为，对于吉非替尼所致肝毒性的患者，换用厄洛替尼，同时加强肝功能监测，是个不错的替代方案。

消化系统不良反应会影响患者的生活质量，降低患者靶向治疗的依从性，增加停药风险，从而影响治疗效果。因此，应加强对患者的宣教，早期发现并识别消化系统不良反应，提前采取相关的预防措施。医生应对患者进行合适的管理，以尽可能降低消化系统不良反应对患者的影响，维持患者的生活质量。EGFR-TKI治疗中应充分权衡抗肿瘤治疗的获益和可能带来的不良反应风险，既避免不必要的剂量减少或者中断治疗从而影响抗癌效果，也避免忽视相关的风险^[24]^。

## 2 EGFR-TKI相关性皮肤不良反应及其处理

EGFR对皮肤生理学有多种作用，包括刺激表皮生长、抑制分化、加速伤口的愈合等。抑制EGFR的活性可导致细胞内信号转导通路的级联反应从而引起多种皮肤不良反应，如皮疹/痤疮样皮疹、皮肤干燥、瘙痒和指甲/甲周组织的炎症^[24]^。EGFR-TKI导致的皮肤不良反应，不仅影响患者生活质量，还可能导致正常治疗无法继续，严重影响肿瘤的治疗效果，其中又以皮疹/痤疮样皮疹和甲沟炎两类不良反应最为常见。因此，本章节着重对皮疹/痤疮样皮疹和甲沟炎两类皮肤不良反应的发生率、高危因素、临床表现、诊断和分级标准以及防治措施进行归纳总结。

### 2.1 不同EGFR-TKI相关性皮肤不良反应的发生率

不同EGFR-TKI的Ⅲ期临床研究中皮疹/痤疮样皮疹的发生率为15.5%-89.1%，≥3级的发生率1%-16.2%^[1, 2, 5, 6, 8-11]^。甲沟炎的发生率4%-56.8%，≥3级的发生率0-11.4%^[1, 2, 5, 6, 8-11]^（表 9）。

**9:** 不同EGFR-TKI的相关Ⅲ期临床研究中皮肤不良反应发生率 Incidence of skin adverse events in phase Ⅲ clinical trials about different EGFR-TKI

EGFR-TKI	研究缩写	地区	皮疹/痤疮样皮疹			甲沟炎	
			所有级别（%）	≥3级（%）		所有级别（%）	≥3级（%）
吉非替尼	IPASS^[1]^	东亚	66.2^*^	3.1^*^		13.5	0.3
	NEJ 002^[2]^	日本	14.9^**^	5.3^**^		文中未提及相关数据	文中未提及相关数据
厄洛替尼	EURTAC^[5]^	欧洲	67^**^	13^**^		文中未提及相关数据	文中未提及相关数据
	OPTIMAL^[6]^	中国	73^**^	2^**^		4	0
阿法替尼	LUX-Lung 3^[8]^	全球	89.1^*^	16.2^*^		56.8	11.4
	LUX-Lung 6^[9]^	亚洲	80.8^*^	14.6^*^		32.6	0
奥希替尼	AURA3^[11]^	全球	34^**^	1^**^		22	0
埃克替尼	CONVINCE^[10]^	中国	15.5^**^	14.9^**^		文中未提及相关数据	文中未提及相关数据
^*^皮疹/痤疮；^**^皮疹							

### 2.2 EGFR-TKI相关性皮肤不良反应的高危因素

由于EGFR存在于所有正常上皮和部分间叶来源的细胞（包括表皮角质形成细胞、外毛根鞘和皮脂腺），因此，EGFR-TKI对皮肤及其附属器官具有特殊的毒副反应^[37]^。EGFR-TKI所致皮肤不良反应的类型和严重程度不仅与所用EGFR-TKI的种类和治疗时间相关，也与患者自身因素相关，如吸烟、免疫状态、遗传变异（如*K*-*ras*突变）等相关^[38]^。加重EGFR-TKI所致皮肤不良反应的因素有阳光暴晒、同期进行放射治疗、皮肤保湿不充分、老年人、曾接受细胞毒药物治疗继而导致皮肤屏障改变的患者等^[37]^。

### 2.3 EGFR-TKI相关性皮肤不良反应的临床症状

EGFR-TKI所致的皮疹/痤疮样皮疹多在靶向药物治疗后1周-2周发生^[39]^，常发生于皮脂腺丰富的部位，严重时下肢亦可受累甚至遍及全身；多伴有瘙痒和皮肤干燥，常使患者心烦意乱，影响日常生活和夜间睡眠^[40]^。EGFR-TKI引起的痤疮样皮疹与寻常痤疮具有差异^[24]^。寻常痤疮是由毛囊不同深度的炎症以及其他继发性反应造成的，初发损害常为白头粉刺及黑头粉刺，伴炎性丘疹、结节和囊肿，常伴有疼痛，皮脂溢出部位好发^[41]^；而EGFR-TKI所致的皮疹形态单一，以丘疹脓疱疹为主，可伴有瘙痒，在临床和组织病理学特征方面都不同于寻常痤疮^[42]^。指甲改变多出现在EGFR-TKI初始治疗后4-8周，可发生于任何指甲或脚趾甲，通常由指（趾）甲根部的边缘开始出现红肿、疼痛，之后两侧甲沟逐渐有发炎、溃疡、出现化脓性肉芽组织等症状，使指（趾）甲内嵌^[42]^，造成患者活动不便。EGFR-TKI所致皮疹和甲沟炎的临床表现见表 10。

**10:** EGFR-TKI导致皮肤不良反应的主要临床表现^[38, 42]^ The main clinical features of skin adverse events in patients treated with EGFR-TKI^[38, 42]^

皮肤不良反应	主要临床表现
痤疮样皮疹	(1) 无黑头粉刺 (2) 常自觉瘙痒 (3) 常发生于面、胸、上背，也可累及任何部位
甲沟炎	(1) 皮疹初起于甲周皮肤 (2) 肉芽组织的形成 (3) 涂片可见革兰氏阳性菌（gram positive bacteria, G^+^）、革兰氏阴性菌（gram negative bacteria, G^-^）以及白色念珠菌感染

### 2.4 EGFR-TKI相关性皮肤不良反应的诊断和分级

根据明确的EGFR-TKI服用史、以及相关的临床症状进行诊断。同时需排除具有类似症状的其他相关疾病^[41]^。

### 2.5 EGFR-TKI相关性皮肤不良反应的分级

对EGFR-TKI皮肤不良反应严重程度的评估是治疗该病的基础。目前通用的分级标准是NCI针对皮肤不良反应制订的分级标准。痤疮样皮疹和甲沟炎的分级参考2017年NCI发布的CTCAE 5.0标准分级^[22]^，具体见表 11。

**11:** 痤疮样皮疹和甲沟炎的分级标准 Grades criteria for acne-like rash and paronychia

分级	皮肤不良反应	
	痤疮样皮疹	甲沟炎
1	丘疹和/或脓疱覆盖 < 10%体表面积（body surface area, BSA），伴或不伴瘙痒和触痛	甲沟肿胀或红斑；甲周皮肤受损
2	丘疹和/或脓疱覆盖10%-30% BSA，伴或不伴有瘙痒和触痛；伴心理影响；日常生活中工具使用受限；丘疹和/或脓疱覆盖 > 30% BSA，伴或不伴轻度症状	需要局部治疗；需要口服给药；甲沟肿胀或红斑伴痛；甲板分离或脱落；日常生活中工具使用受限
3	丘疹和/或脓疱覆盖 > 30% BSA伴中度或重度症状；生活自理受限；伴局部超感染，需要局部抗生素治疗	需要手术治疗；需要静脉抗生素治疗；日常生活自理能力受限
4	威胁生命；丘疹和/或脓疱累及任意体表范围，伴或不伴有瘙痒或触痛，与广泛超感染有关，需要静脉抗生素治疗	原文中未提及
5	死亡	原文中未提及

### 2.6 EGFR-TKI相关性皮肤不良反应的预防

使用EGFR-TKI治疗之前，医护人员应向患者及其家属进行患者教育^[43]^：首先，EGFR-TKI所致的皮疹不具有传染性；其次，皮疹与普通痤疮具有差别，部分非处方药（over the counter, OTC）类的痤疮治疗药物缺乏疗效^[43]^。指导患者采取正确的预防措施，如嘱咐防晒，建议使用防晒系数（sun protection factor, SPF≥30）的广谱防晒用品^[24]^；每天保持皮肤的清洁与湿润，温水洗浴后适当涂抹保湿乳霜^[24]^；EGFR-TKI治疗过程中需穿宽松、透气的鞋子，坚持温水沐足后涂抹润肤霜可预防足部皮疹的发生，治疗足癣等原发疾病^[24]^。

对于不同类型的皮肤不良反应的预防措施见表 12。

**12:** EGFR-TKI相关性痤疮样皮疹和甲沟炎的预防措施 Prevention measures of acne-like rash and paronychia in patients treated with EGFR-TKI

皮肤不良反应	预防措施
痤疮样皮疹	使用SPF≥30的防晒霜、滋润霜^[24]^；仔细的皮肤护理^[42]^
甲沟炎	保持手部和足部的皮肤干燥、不要将手和脚浸泡在肥皂水中，经常使用滋润霜；避免指甲受伤；穿鞋前确保脚部干燥；修剪指甲时要小心；戴棉手套；穿宽松、舒适的鞋子保护趾甲；避免皮肤受刺激^[24]^
SPF：防晒系数。国外对痤疮样皮疹，推荐预防性用药（服用靶向药物起6周）：口服多西/米诺环素（100 mg *bid*）+外用低效糖皮质激素/克林霉素凝胶（5A）^[42]^或根据经验外用夫西地酸软膏（5B）	

### 2.7 EGFR-TKI相关性皮肤不良反应的处理

针对痤疮样皮疹和甲沟炎的具体治疗方法见表 13和表 14。

**13:** EGFR-TKI相关性痤疮样皮疹的治疗 Treatment of acne-like rash in patients treated with EGFR-TKI

分级	治疗措施
1级	(1) 外用2.5%氢化可的松霜剂及抗生素，可选择的抗生素有：1%克林霉素凝胶（5A）^[42]^，或硫酸新霉素（5A）^[24]^，或1%的甲硝唑（5A）^[44]^或根据经验使用夫西地酸软膏（5B）；评估2周，如病情无改善则按下一级处理^[43]^； (2) 如果伴有瘙痒，可酌情使用一代或二代抗过敏药，一代抗过敏药如扑尔敏（5B）、酮替芬（5B）、赛庚啶（5B）等因具有镇静嗜睡作用^[45]^更适用于有夜间瘙痒的患者
2级	(1) 在1级治疗措施的基础上，加用他克莫司软膏（5A），口服多西环素或米诺环素（100 mg *bid*）（5A）^[42]^； (2) 评估2周，如病情无改善则按下一级处理^[42]^
3级	(1) 按照说明书调整靶向药物剂量^[42]^ (2) 必要时需行细菌/真菌/病毒培养；除维持2级治疗外，需加用强的松（0.5 mg/kg/d）×5天（5A）；评估2周，如病情无改善，则需停用靶向药物^[42]^；停药后继续治疗皮疹，必要时可咨询皮肤科医生。当皮疹恢复至≤2级，可重新使用EGFR-TKI（减少剂量），治疗同2级治疗，必要时口服抗生素和局部使用糖皮质激素^[24]^； (3) 顽固性瘙痒可酌情使用加巴喷丁或普瑞巴林等药物（5A）^[44]^
4级	(1) 4级治疗措施同3级 (2) 停用靶向药物

**14:** EGFR-TKI相关性甲沟炎的治疗 Treatment of paronychia in patients treated with EGFR-TKI

分级	治疗措施
1级	(1) 外用抗生素（克林霉素，夫西地酸，百多邦）及白醋浸泡（5A）（手浸泡含1：1白醋与水的混合液，每天15 min）^[42]^； (2) 评估2周，如病情无改善则按下一级处理^[42]^； (3) 必要时还需外用强效的糖皮质激素和抗生素/抗真菌药物，如0.05%丙酸氯倍他索（5A）、0.3%戊酸二氟米松（5A）、硫酸新霉素（5A）^[24]^、酮康唑（5B）、联苯苄唑乳膏（美克）（5B）、特比萘芬乳膏（兰美抒）（5B）等。
2级	(1) 除1级治疗外，需加用每日外用1次碘酊（5A）； (2) 评估2周，如病情无改善则按下一级处理。
3级	(1) 按照说明书调整靶向药物剂量；必要时需行细菌/真菌/病毒培养^[42]^； (2) 口服抗生素（如多西环素100 mg/d）治疗（5A），必要时拔甲； (3) 评估2周，如病情无改善，则需停用靶向药物^[42]^； (4) 停药后继续治疗甲沟炎，必要时可咨询皮肤科医生； (5) 当甲沟炎恢复至≤2级，可重新使用EGFR-TKI（减少剂量），治疗同2级治疗； (6) 持续使用外用强效的糖皮质激素和抗生素/抗真菌药物，如0.05%丙酸氯倍他索（5A）、0.3%戊酸二氟米松（5A）、硫酸新霉素（5A）^[24]^、酮康唑（5B）、联苯苄唑乳膏（美克）（5B）、特比萘芬乳膏（兰美抒）（5B）等。

国外有文献推荐：治疗痤疮样皮疹1级时，可使用1%-2%的红霉素^[43]^；治疗2级痤疮样皮疹时，可口服土霉素500 mg *bid*（5A）^[44]^。

治疗瘙痒时，可使用的抗组胺药包括：左西替利嗪5 mg *qid*、地氯雷他定5 mg *qid*、苯海拉明25 mg-50 mg *tid*、羟嗪25 mg *tid*或非索非那定60 mg *tid*。重度瘙痒者可使用γ-氨基丁酸（γ-aminobutyric acid, GABA）受体激动剂（如果患者有肾功能损害则调整），如加巴喷丁每8小时300 mg或普瑞巴林每8小时50 mg-75 mg；三环类药物，如多西平每8小时25 mg-50 mg、阿瑞匹坦三次：第1天125 mg，第2天和第3天各80 mg（5A）^[44]^。

国外推荐用药：治疗1级甲沟炎时，可外用1%红霉素、1%四环素、1%氯霉素和碘软膏和20%硝酸银（5A）^[44]^。

总之，多数EGFR-TKI所致痤疮样皮疹和甲沟炎是可防、可控的，亦有部分患者的皮疹随治疗而趋向稳定，相对严重的患者停药后皮疹可消退，再次用药皮疹再发或加重。因此为改善患者生活质量，提高靶向治疗的依从性，应正确防治EGFR-TKI所致的痤疮样皮疹和甲沟炎。对轻-中度不良反应，可采取综合防治措施如生活方式及药物干预等提高患者的生活质量，而无需调整靶向药物的剂量或中断治疗从而影响抗癌效果；对重度不良反应，应系统用药对症改善症状，必要时减量或暂停抗肿瘤靶向治疗，待皮疹改善后调整抗癌方案^[37]^。

### 2.8 EGFR-TKI相关性皮疹的中医论治

#### 2.8.1 病因病机

EGFR-TKIs相关性皮疹即药疹，祖国医学又称为“药毒疹”。关于皮疹的病因病机，《素问·生气通天论》中有“汗出见湿，乃生座雍”；“劳汗当风，寒薄为皶，郁乃痤”的描述，意思是邪气郁于肌肤腠理而致皮疹。《素问·评热病论》中有：“邪之所凑，其气必虚”。中医理论认为，发病的病因病机总由禀赋不耐、邪毒侵犯，复因感受药物特殊之毒所致。外邪侵袭腠理，内不得疏泄，外不得透达，郁而化热，耗血伤阴，血虚生风化燥，肌肤失养；或禀血热之体，受药毒侵扰，火毒炽盛，燔灼营血，外发皮肤，内攻脏腑；或禀湿热之体，受药毒侵扰，体内湿热蕴蒸，郁于肌肤；病久药毒灼伤津液，气阴两伤，肌肤失养。本病由药毒及外界之虚邪贼风所致，概而言之，主要为“风、热、湿、毒、瘀”等邪气郁于皮肤故而发为药疹。

#### 2.8.2 辨病辨证

EGFR-TKI引起的皮损以痤疮样皮疹为主，其表现形式类似于寻常痤疮，均表现为面部、头颈部、胸背部多发丘疹或脓疱疹，此类皮疹常伴有瘙痒、干燥和指甲改变。

中医药治疗应从皮损外观、虚实、症状及脏腑病位等四个方面辩证论治，分述如下。

##### 2.8.2.1 辨皮损

###### 2.8.2.1.1 辨形态

如丘疹高出皮面，可伴毛囊角化；如皮疹色红多为血分有热，颜色晦暗则为气滞血瘀；若伴渗液多为湿热流注，若伴瘙痒难忍多从风热辨治；若仅见红斑，不隆起、不凹陷多从风邪袭表辨治；若有水疱则多为湿热、热毒；若有脓疱，多为痰湿、热毒炽盛；亦有皮疹呈结节样，多为气血凝滞。

###### 2.8.2.1.2 辨颜色

若皮疹色红，且压之褪色，多为血热、风热，如若压之不褪色，为兼夹血瘀；若其色淡白，多为气血凝滞，或血虚风邪。

###### 2.8.2.1.3 辨光泽

若色红鲜润，则为阳证；若色泽晦暗，则辨为阴证。

###### 2.8.2.1.4 辨渗液

皮疹若属干型皮损，多会有脱屑、瘙痒等症状，辩证多从其夹风论治；若为湿型皮损，则会有水疱、渗液等症状，需从夹湿论治。

##### 2.8.2.2 辨虚实

###### 2.8.2.2.1 实证

实证包括湿、热、瘀毒等证，若为湿证，多有水疱、渗液等症状，故以利湿为法；若为热证，则多见红肿、灼热、疼痛等症状，治当以清热为法；若为瘀毒，其多有瘀血表现，当以祛瘀解毒为法治疗。

###### 2.8.2.2.2 虚证

虚证主要以血虚为主，多表现为皮肤干燥、皮疹淡白，治以养血补血为法；若其病夹风，则会有皮屑，瘙痒等症状，当以祛风为法；若夹痰，其皮疹多有脓疱，应当化痰解毒。

##### 2.8.2.3 辨症状

###### 2.8.2.3.1 辨痒

若皮肤瘙痒，多为风湿热等邪气客于皮肤，致气血不和；风痒多表现为瘙痒游走不定，时作时休，泛发、起病迅速；若为湿痒其皮疹伴水疱糜烂、渗液；热痒则皮疹色红，瘙痒伴有疼痛；若属血虚风燥，皮肤失于濡养，则瘙痒伴皮肤干燥、脱屑，日久皮肤硬厚。

###### 2.8.2.3.2 辨痛

疼痛可分寒邪、热邪、气滞、痰凝、血瘀阻滞，主因为“不通则痛”。寒邪多见皮肤苍白或青紫，遇寒加重，得温缓解；热邪多见皮肤发红、灼热，疼痛；气滞则表现为胀痛，疼痛程度常随情志变化；痰凝可伴有结节、脓液，血瘀则其疼痛固定不移，皮损呈结节或肿块，初起隐痛、色红，后可转为青紫肿胀。

###### 2.8.2.3.3 辨感觉

灼热感多为热邪蕴结、炙灼皮肤；蚁行感则为气血失和；麻木感多由气血凝滞不通、痰湿瘀血阻滞经络，皮肤失于濡养所致。

##### 2.8.2.4 辨脏腑病位

脏腑气机与皮疹的发病密切相关，本病多与肺、脾、肝、肾有关，《灵枢》言：“肺主皮毛”，皮毛由肺输布的卫气和津液滋养，肺主宣发，调节汗孔开合和排泄汗液。若肺气虚，固护肌表功能障碍，易感外邪；若肺气郁闭，则邪气易滞于皮肤，发为皮疹。另外，脾与皮疹的发生亦关系密切，在《素问·至真要大论篇》说：“诸湿肿满，皆属于脾”，脾胃运化输布水谷精微，脾虚不能主导运化功能而出现水湿停留、肿胀。若患者有肝肾亏虚，易致阴血不足，肌肤失于滋养，可见皮肤干燥、脱屑等症状。

#### 2.8.3 辨证分型

根据中医理论及临床经验，皮疹主要分以下四个证型。

##### 2.8.3.1 肺经风热证

证候：面部丘疹色红，或有脓疱，或有痒痛，伴口渴喜饮，大便干结、小便短赤，舌红苔薄黄，脉弦滑。

##### 2.8.3.2 肠胃湿热证

证候：颜面、胸背皮肤油腻，皮疹红肿疼痛，或有脓疱，伴有口臭，便秘，小便黄，舌红苔黄腻，脉滑数。

##### 2.8.3.3 阴虚内热证

证候：面部皮疹以红色或皮色粉刺丘疹为主，或伴有小脓疱、小结节。口干、心烦、失眠多梦、大便干结、小便短赤。舌红少苔或薄黄苔，脉数或细数。

##### 2.8.3.4 瘀热痰结证

证候：皮疹多颜色暗红，皮损以结节、脓疱、囊肿和凹凸不平的瘢痕为主，经久难愈，或伴有纳呆腹胀。舌红或暗红有瘀点，苔黄腻，脉弦滑或弦细。

#### 2.8.4 治则治法

余等^[46]^以肺肾阴虚论其本，以内外合邪论其标，结合温病学卫气营血理论解读其发展演化，认为EGFR-TKI类药疹的根本病机乃阴虚血燥在内而毒邪结聚在外，治疗上宜以养阴润燥以扶其本虚，再根据病邪的不同阶段以宣肺、清热、凉血、化瘀以解其标实，将皮疹患者分为肺经风热、肠胃湿热、阴虚内热、瘀热痰结四证进行辨证论治，内服以经验方加减荆防四物汤化裁，随证加减，并结合消疹止痒汤外洗，双管齐下，在临床工作中取较好疗效，并为中西医结合治疗提供思路，可供临床工作者学习借鉴。

##### 2.8.4.1 主方

加减荆防四物汤：

【组成】荆芥10 g、防风10 g、生地黄20 g、赤芍10 g、当归10 g、川芎10 g、白鲜皮15 g、紫草10 g、蝉蜕10 g、甘草6 g。

【用法】水煎至250 mL，分温内服。

【功效】祛风清肺，凉血润燥。

【主治】风热阴虚血燥之皮疹。症见丘疹色红，或有脓疱，或有小结节，或有痒痛，伴口渴喜饮，或见心烦、失眠多梦，大便干结、小便短赤，舌红苔薄黄，脉弦、细数。

血虚者：加用鸡血藤30 g、熟地黄30 g等。

风盛者：加用薄荷10 g（后下）、白蒺藜30 g、飞扬草30 g、地肤子30 g等。

痰盛者：加用桑叶15 g、连翘10 g、猫抓草15 g、浙贝母15 g等。

血瘀者：加用莪术10 g、红花10 g、牡丹皮30 g等。

热盛者：加用金银花30 g、蒲公英30 g、黄芩10 g、黄连10 g等。

湿重者：加用绵茵陈30 g、薏苡仁30 g、白术15 g、法半夏15 g等。

##### 2.8.4.2 外洗

消疹止痒汤：

【组成】黄柏30 g、苦参30 g、徐长卿30 g、地肤子30 g、白鲜皮30 g、百部30 g、山楂30 g、乌梅30 g、当归30 g、飞扬草30 g。

【用法】水煎至500 mL-1, 000 mL，外洗患处。

【功效】清热凉血，祛瘀止痒。

【主治】各种瘙痒性皮疹。根据具体情况加减，瘙痒严重者，可加蝉衣30 g、薄荷30 g（后下）；热毒较甚者，可加连翘30 g、蒲公英30 g、金银花30 g等；皮疹色暗，皮损经久难愈者，可加乳香30 g、没药30 g、五倍子30 g等。

#### 2.8.5 中医各家学说

随着靶向药物的广泛使用，出现EGFR-TKI所致皮疹的患者也在增多，许多中医临床研究报道了中医药对于口服EGFR-TKI所致皮疹有着良好的治疗效果。

石等^[47]^发现银翘散加减治疗吉非替尼引起的皮疹同样有一定疗效[治疗组有效率为100%，对照组为80%（*P* < 0.01），2b级]。张等^[48]^发现养肺消疹方对肺癌靶向药物所致皮疹有较好的临床疗效[治疗组总有效率为75% vs对照组总有效率为55%（*P* < 0.01），2b级]。朱等^[49]^加味消风散治疗吉非替尼所致皮疹也具有较好的临床疗效[总有效率治疗组与对照组分别为93.75%、62.50%（*P* < 0.05），2b级]；且消风散治疗吉非替尼引起痤疮样皮疹疗效优于一般类固醇药物，不良反应较少。张等^[50]^研究报道LG09外敷方治疗NSCLC患者口服EGFR-TKI所致皮疹的疗效显著[治疗组有效率86.67% *vs* 对照组63.33%（*P* < 0.01），2b级]。邓等^[51]^发现清肺排毒凉血中药内服结合氢化可的松及红霉素软膏外涂用于治疗吉非替尼相关性皮疹疗效良好[治疗组、对照组有效率分别为85.0%、41.7%（*P* < 0.05），2b级]。彭等^[52]^等应用“止痒平肤液”（黄芩、苦参、白鲜皮、马齿苋等）湿敷或浸洗患处，对照组采用治疗后皮肤瘙痒、皮疹以及脱屑等皮肤毒性均有改善（*P* < 0.05，2b级），从而明显改善患者的生活质量。

## 3 EGFR-TKI相关性口腔粘膜炎及其处理

EGFR-TKI类药物在治疗中可引起药物相关口腔粘膜炎等不良反应。现就EGFR-TKI类药物会导致药物相关口腔粘膜炎的发生率、分级、临床表现、危险因素、预防和治疗阐述如下。

### 3.1 EGFR-TKI相关性口腔粘膜炎的发生率

不同的EGFR-TKI药物所致的口腔粘膜炎其发生率及严重程度存在一定差异。其中，吉非替尼所致所有级别口腔粘膜炎的发生率约17%-23.9%，3级及以上的发生率 < 1%^[1, 53]^；厄洛替尼所致的口腔粘膜炎的发生率约8%-13%，3级及以上的发生率为1%^[6, 54]^；阿法替尼所致口腔粘膜炎的发生率约51.9%-72.1%，3级及以上的发生率4.4%-8.7%^[8, 9, 53]^；奥希替尼所致口腔粘膜炎的发生率约15%-29%，3级及以上的发生率 < 1%^[11, 55]^。表 15为这些Ⅲ临床研究所示的口腔粘膜炎的发生率。

**15:** 不同EGFR-TKI的相关Ⅲ期临床研究中口腔粘膜炎的发生率 Incidence of stomatitis/mucositis in phase Ⅲ clinical trials about different EGFR-TKI

EGFR-TKI	研究缩写	区域	口腔粘膜炎	
			所有(%)	≥3级%
吉非替尼	IPASS ^[1]^	东亚	17	0.2
	NEJ 002^[2]^	日本	文中未提及相关数据	文中未提及相关数据
厄洛替尼	EURTAC^[5]^	欧洲	文中未提及相关数据	文中未提及相关数据
	OPTIMAL^[6]^	中国	13	1
阿法替尼	LUX-Lung3^[8]^	全球	72.1	8.7
	LUX-Lung 6^[9]^	亚洲	51.9	5.4
奥希替尼	AURA3 ^[11]^	全球	15	0
埃克替尼	CONVINCE ^[10]^	中国	文中未提及相关数据	文中未提及相关数据

### 3.2 EGFR-TKI相关性口腔粘膜炎的临床表现、诊断、分级

EGFR-TKI药物引起的口腔粘膜炎，常在用药开始第13-19天出现^[56]^。病人口腔粘膜出现红斑、水肿、糜烂，进一步形成点状、片状溃疡，可波及上下唇、双颊、舌、口底粘膜；粘膜溃疡表覆伪膜、渗血，引起疼痛、吞咽困难、味觉异常等。口腔粘膜炎分级亦采用CTCAE 5.0的分级标准，见表 16^[22]^。

**16:** 口腔粘膜炎的分级标准 Grades criteria for stomatitis/mucositis

分级	描述
1	无症状或轻微症状，无需治疗
2	中度疼痛或溃疡，不影响经口进食，需调整饮食
3	严重疼痛，影响经口进食
4	危及生命，需紧急治疗
5	死亡
口腔粘膜炎定义：口腔粘膜出现溃疡或炎症。	

### 3.3 EGFR-TKI相关性口腔粘膜炎的危险因素及造成的危害

见表 17。

**17:** EGFR-TKI相关性口腔粘膜炎危险因素及危害 Risk factors and harm of stomatitis/mucositis in patients taking EGFR-TKI

危险因素	危害
口腔卫生差	口腔软垢、牙石堆积、牙齿松动、龋齿、残根、残冠、慢性根尖周炎反复发作^[57]^
义齿	不良修复体刺伤粘膜^[58]^
高龄	因唾液流量减少造成粘膜角化层薄，上皮细胞更新能力降低，粘膜愈合延缓^[59, 60]^
酒精和烟草摄入	长期刺激造成粘膜慢性炎症^[60]^
吸氧口呼吸	口腔粘膜干燥易受损^[60]^
服用抗胆碱能、组织胺、类固醇药物	粘膜干燥真菌过度生长^[60]^
热、酸、粗糙食物	刺激损伤粘膜^[60]^
营养不良	精制糖增加龋齿；蛋白质/热量摄入不足使愈合延迟；维生素缺乏引起口腔并发症^[60]^
脱水	口腔粘膜干燥或干裂^[60]^

### 3.4 EGFR-TKI相关性口腔粘膜炎的预防

鉴于当前国人口腔卫生健康状况不容乐观，龋病、牙周炎及口腔粘膜病发病率高，目前仍是常见的口腔疾患^[61]^。所以建议在使用EGFR-TKI类药物之前，应接受口腔健康教育指导^[59]^，从而降低EGFR-TKI相关口腔粘膜炎的发生率和级别^[58, 62]^。

口腔健康教育的内容包括：指导肿瘤病人完成日常个性化的口腔卫生维护，包括口腔保健品（牙刷、牙膏、牙线、牙缝刷、冲牙器）的选择及使用，如发现病人口腔存在严重感染病灶应适度干预^[59]^。与病人建立联系，以便对使用EGFR-TKI相关药物的病人进行随诊，及时发现并干预1级和2级口腔粘膜炎，适时适度缓解症状，防止口腔粘膜炎发展成3级及以上。

### 3.5 EGFR-TKI相关性口腔粘膜炎的治疗

EGFR-TKI所致的口腔粘膜炎，其临床处理原则及目的为：控制疼痛，覆盖溃疡面，使其尽早愈合；保持口腔清洁，减少多重感染；阻止口腔粘膜炎发展为3级或4级；多学科协作治疗口腔粘膜炎引起的溃疡出血、口腔多重感染、营养不良、脱水、电解质紊乱等并发症。EGFR-TKI类药物治疗前及治疗中，肿瘤专科医师、口腔医师、营养师应沟通协作；口腔医护人员指导患者自用药起，每天自行完成口腔检查、口腔清洁，保证每日均衡营养及水的摄入，禁烟酒，禁用含有酒精的含漱剂，唇部干燥可使用无刺激性油膏；如有不适，可与口腔医师沟通^[63]^。见表 18。

**18:** EGFR-TKI相关性口腔粘膜炎管理措施 Management of stomatitis/mucositis in patients treated with EGFR-TKI

分级	治疗措施
1级	（1）如溃疡疼痛影响进食，可在进食前使用利多卡因溶液、利多卡因凝胶或苯佐卡因糊剂涂布于溃疡处^[58, 64]^（5B）； （2）进食少渣、滑润食物，避免酸、热、辛辣食物^[24, 58]^（5B）； （3）每天进餐后即刻口腔清洁，使用小头软毛牙刷，刺激性小的牙膏。餐后使用4%碳酸氢钠含漱剂或0.12%氯己定含漱剂，每次10 mL，含漱3 min-5 min^[59, 65]^（4A）。之后可使用0.1%曲安奈德口内膏涂布于溃疡处，3次/天，促进愈合^[58, 66]^（5B）。
2级	在1级治疗的基础上： （1）如口腔粘膜干燥可使用人工唾液，口腔湿润凝胶^[59, 67]^（5B），保持室内湿度适宜，保证每日水的摄入量； （2）观察口腔是否发生多重（细菌、真菌、病毒）感染^[24]^； （3）使用低能量激光照射溃疡处，5天/周，加速溃疡愈合^[59, 65, 68]^（2A）。
3级	（1）与经治医师沟通是否EGFR-TKI药物减量^[24]^； （2）请临床营养师制定个性化膳食，摄入流食或半流食，防止呛咳； （3）如严重疼痛影响生活质量，可全身给予止痛剂和抗焦虑药物：吗啡、芬太尼、多虑平^[69]^（2A）； （4）口腔真菌感染可口服制霉菌素50万u/片，1片/次，3次/天，7天；或氟康唑100 mg/d-200 mg/d，服用2周；单纯疱疹病毒感染引起的口角炎可使用阿昔洛韦乳膏3次/天，涂布双口角，如口腔粘膜出现大范围病毒感染病损可口服阿昔洛韦200 mg/d-800 mg/d，3次/天，3天-5天，或伐昔洛韦500 mg/次，2次/天^[70]^（5B）；如口腔粘膜炎经治疗恢复至≤2级，与经治医师沟通，可重新使用EGFR-TKI药物。
4级	（1）与经治医师沟通停用EGFR-TKI药物^[24]^； （2）被动口腔清洁，护理人员完成口腔基础护理2次/天-3次/天； （3）结合病人情况，可使用全身止痛药和抗焦虑药，如吗啡、芬太尼、多虑平等^[68, 69]^； （4）控制口腔多重感染； （5）警惕因深大溃疡引起口腔粘膜、牙龈渗血并止血^[61]^； （6）必要时实施肠外营养治疗^[59]^（5B）。

国外指南或文献对口腔粘膜炎的管理措施和用药推荐：

1级：餐后可使用苄达明含漱液^[71]^（1A）。可使用帕利夫明（human keratinocyte growth factor-1 palifermin）^[68]^（2A）。

3级：如严重疼痛影响生活质量，可局部给予2%吗啡含漱剂（3A）、0.5%多虑平含漱剂（4A）^[59, 67]^。

### 3.6 EGFR-TKI相关性口腔粘膜炎的中医论治

EGFR-TKI相关性口腔溃疡属中医“口疮”的范畴。明代薛己《口齿类要·口疮》中记载：“口疮，上焦实热，中焦虚寒，下焦阴火，各经传变所致，当分别而治之”。上焦实热多为心脾积热；下焦阴火为肾阴亏虚，虚火上炎；中焦虚寒为脾肾阳虚。由此可见，口疮病位关乎心、脾、肾。

#### 3.6.1 辨病辨证

##### 3.6.1.1 辨虚实

口疮表面覆盖黄色、黄白色假膜，周边红肿，灼痛明显者，为实热；口疮面积小，个数少，色灰白，周边红肿不甚，疼痛较轻，为虚热；口疮色白或暗，周边淡红或不红，疼痛轻，多为阳虚。

##### 3.6.1.2 辨病程长短

口疮起病急者，多为实热；口疮反复，此愈彼起，延绵不止，多为虚热；口疮久难愈合，迁延不愈，多为阳虚。

#### 3.6.2 辨证分型

根据中医理论及临床经验，其证型主要有以下三个^[72]^。

##### 3.6.2.1 心脾积热证

口为脾之窍，舌为心之苗，心脾积热，上炎口舌。症候：口腔粘膜溃疡，灼热疼痛明显，伴口干口苦，心烦失眠，大便秘结。舌红，苔黄或腻，脉数有力。

##### 3.6.2.2 阴虚火旺证

肾阴亏虚，相火无制，上炎口舌。症候：口腔粘膜溃疡数量较少，疼痛较轻，反复发作，伴手足心热，失眠多梦，或口干不欲饮，夜间盗汗。舌红少苔，或有裂纹，脉细数。

##### 3.6.2.3 阳虚寒湿证

症候：口腔粘膜疼痛不明显，色淡暗，经久不愈；伴倦怠乏力，面色㿠白，或腰膝酸冷，大便溏烂，小便清长。舌淡，苔白，脉沉迟或弱。

#### 3.6.3 治则治法

EGFR-TKI所致口腔溃疡的病机，多为心脾积热，或虚火上炎，熏发口舌而致。因此，治疗上宜以清心泻火，佐以养阴生津，并随证加减。

##### 3.6.3.1 主方

清胃散加减：

【组成】生地黄20 g、当归10 g、牡丹皮15 g、黄连5 g、灯芯草3 g、桑叶15 g、升麻10 g

【用法】水煎至250 mL，分温内服。

【功效】清心泻火，凉血养阴。

【主治】心脾积热之口疮，症见口疮灼痛明显，伴口干口苦，舌红苔黄或腻，脉滑数。

热盛伤阴者：加用玄参15 g、天花粉15 g、蒲公英30 g、连翘15 g等以清热养阴。

兼见大便秘结者：加用大黄10 g、芒硝10 g以泻热通便、导热下行。

兼见心烦失眠者：加用栀子15 g、竹叶15 g以清心除烦。

心肾阴虚者：加用鳖甲30 g、龟板30 g、北沙参30 g、麦冬15 g以滋阴养血。

虚火上炎者：可加用肉桂3 g反佐，或牛膝15 g以引火归原。

脾肾阳虚者：上方去黄连、灯芯草、桑叶，加用黄芪30 g、党参25 g以健脾益气，白术15 g、茯苓25 g以健脾化湿，干姜10 g、熟附子10 g以温肾散寒。

##### 3.6.3.2 含漱方

口炎含漱液：

【组成】黄连5 g、黄芩5 g、大黄5 g、黄柏5 g、栀子5 g、薄荷5 g、桔梗5 g、甘草5 g、乌梅5 g、金银花10 g。

【用法】沸水泡开，凉后含漱。

【功效】清热解毒、散结利咽。

【主治】各种口腔溃疡。根据具体情况加减，痰瘀互结者，加陈皮5 g、桃仁5 g；溃疡日久不愈者，加白芨5 g、白芷5 g；疼痛较甚者，加土牛膝5 g、射干5 g；咽痒者，加蝉衣5 g、僵蚕5 g。

#### 3.6.4 中医各家学说

中医药针对EGFR-TKI相关性口腔溃疡的研究报导较为缺乏，但从中医理论角度分析，其他药物所致口腔溃疡与EGFR-TKI相关性口腔溃疡辨证思路相同，其病机多关乎实热、阴虚、气虚，而治疗亦多从清热、养阴、补气着手。因此，此部分将列举中医药治疗口腔溃疡的相关研究，以开拓EGFR-TKI相关性口腔溃疡的中医治疗思路。

张等^[73]^认为化疗后口腔溃疡属湿热蕴结、灼血伤络、心肾不交、气滞血瘀之证，宜选用清热解毒、凉血消斑、利咽消肿、解毒散结、活血化瘀类药物进行治疗，临床以青参甘汤（大青叶15 g、玄参15 g、甘草5 g，每1剂煎成500 mL含漱液）治疗化疗后湿热毒蕴证口腔溃疡，其有效率95.97%，高于对照组（0.02%呋喃西林和1%-3%过氧化氢溶液交替含漱）69.40%，其恢复普食时间、体温恢复正常时间、口腔溃疡恢复时间亦短于对照组。

康复新液由大蠊干燥虫体提取物质制成，具有通利血脉，养阴生肌的功效；夏等^[74]^对300例口腔溃疡患者进行分组，试验组予康复新液口含及服用治疗，对照组予碘甘油涂布，结果发现与对照组相比较，试验组的溃疡持续时间、疼痛指数明显降低（*P* < 0.05），表皮生长因子（epidermal growth factor, EGF）、EGFR情况改善（*P* < 0.05），提示康复新液治疗口腔溃疡能够缩短溃疡持续时间，降低疼痛指数，促进EGF、EGFR分泌，恢复口腔粘膜细胞的修复能力。

涂等^[75]^用滋阴扶正方加减方（太子参15 g、黄芪30 g、白术15 g、女贞子20 g、黄精30 g、玄参15 g、生地15 g、淡竹叶10 g、黄连6 g、甘草6 g，每日1剂，水煎口服）预防化疗所致阴虚为主的口腔粘膜炎，其口腔溃疡发生率、平均病程均低于对照组（生理盐水100 mL+维生素B_12_ 100 mg+制霉菌素50万U配制的漱口液）。

耿等^[76]^认为恶性肿瘤患者素体气血亏虚，化疗药物进一步克伐脾胃，导致气血生化运行不畅，“脾气通于口”，虚火上炎，则易致口疮，其用补中益气汤加减治疗肿瘤化疗后口腔溃疡取得显著疗效，2周后试验组总有效率高于康复新液组。

此外，使用EGFR-TKI治疗的患者在用药期间，在日常生活中需积极预防与调护，以减轻EGFR-TKI相关性口腔溃疡的发生。如注意口腔卫生，早晚刷牙，饭后漱口；避免进食硬物，以防损伤口腔粘膜；佩戴义齿者，需注意义齿的机械刺激损伤粘膜。而对于已发生口腔溃疡的患者，饮食亦需加注意，如实火口疮者，忌食辛辣刺激、肥甘厚味之物；虚火口疮者，忌食生冷寒凉之物，同时颐养心性，戒忧思、恼怒。不宜过劳，避免劳累或熬夜损伤正气。

## 4 EGFR-TKI相关性间质性肺疾病ILD及其处理

ILD是以肺间质为主要病变的众多异质性疾病的总称，以局灶或弥漫性肺间质的非感染性炎性改变和进行性纤维化，甚至发展为呼吸衰竭和心功能不全为病变特点^[77, 78]^。EGFR-TKI导致的ILD虽然发生率较低，但一旦发生可严重威胁患者的生命^[79]^。

EGFR-TKI相关性ILD的发生机制，可能是因为肺泡Ⅱ型上皮细胞表达EGFR，参与肺泡壁的修复，而EGFR-TKI和抗EGFR单抗二者在抑制肿瘤生长的同时，也抑制了气管上皮细胞的生长和损伤的修复而加重肺损害。此外，EGFR-TKI可能引起肺泡和支气管上皮损伤及慢性炎症，二者均刺激成纤维细胞迁移、增生、产生细胞外基质，从而引起肺纤维化，导致ILD的形成^[80, 81]^。另外，部分ILD可能是由于机体对EGFR-TKI的免疫介导的过敏反应所导致^[81]^。以下对EGFR-TKI所致ILD的发生率、高危因素、诊断和分级标准以及防治措施进行归纳总结。

### 4.1 不同EGFR-TKI相关性间质性肺疾病发生率和死亡率

不同EGFR-TKI的Ⅲ期临床研究中药物相关性ILD的发生率和死亡率不同（发生率：0%-5.3%；死亡率0%-0.9%）^[1, 2, 5, 6, 8-11]^，见表 19。此外，不同EGFR-TKI相关性ILD的发生时间也不同，见表 20。

**19:** 不同EGFR-TKI的相关Ⅲ期临床研究中ILD的发生率和死亡率 Incidence of ILD in phase Ⅲ clinical trials

EGFI-TKI	研究缩写	区域	ILD（所有级别)	死亡率（%）
吉非替尼	IPASS^[1]^	东亚	2.6^*^	0.5
	NEJ 002^[2]^	日本	5.3^*^	0.9
厄洛替尼	EURTAC^[5]^	欧洲	1^*^	0
	OPTIMAL^[6]^	中国	0	0
阿法替尼	LUX-Lung 3^[8]^	全球	1^*^	0
	LUX-Lung 6^[9]^	亚洲	0.4^**^	0
奥希替尼	AURA 3^[11]^	全球	4^*^	0.4
埃克替尼	CONVINCE^[10]^	中国	0	0
^*^间质性肺疾病；^**^间质性肺炎；†肺炎；ILD：间质性肺疾病				

**20:** 不同EGFR-TKI相关性ILD发生时间 Time of ILD in patients treated with EGFR-TKI

研究	样本量（ILD患者人数/总样本量）	治疗药物	ILD发生时间的范围	ILD发生时间的平均值或中位值
Kataoka（2006）^[83]^	4/489	吉非替尼	14 d-27 d	平均15 d
Cohen（2003）^[84]^	408/50, 005	吉非替尼	大多数发生在服药后4周内	中位时间为24 d-42 d
Nakagawa（2012）^[85]^	158/3, 488	厄洛替尼	5天-9个月	中位时间为39 d
Nobuyuki Yamamoto^[86]^	60/1, 602	阿法替尼	3 d-329 d	中位时间为35.5 d
奥西替尼说明书^[87]^		奥希替尼	大多数发生在3个月以后	中位时间2.7个月
ILD：间质性肺疾病				

### 4.2 EGFR-TKI相关性间质性肺疾病的危险因素

EGFR-TKI相关性ILD的危险因素包括：男性，近期放化疗史；吸烟史；年龄≥55岁；体力状态（performance status, PS）评分 > 2分；影像学检查显示正常肺组织 < 50%；有间质性肺疾病病史；肺气肿或慢性阻塞性肺病；肺部感染；被诊断为癌症的时间短（< 6个月）；合并心血管疾病^[88, 89]^。

### 4.3 EGFR-TKI相关性间质性肺疾病的临床表现

EGFR-TKI相关性ILD起病方式多样，既可表现为用药数天至数周即有明显临床表现的急性或亚急性起病，甚至短期内危及生命，也可表现为慢性隐匿起病，逐渐进展至呼吸衰竭，发现时已属不可逆转阶段。症状、影像和病理表现各异，有时与肿瘤进展难以鉴别，少数情况下可因合并感染而加重病情，或因需治疗EGFR-TKI相关性ILD而终止抗肿瘤治疗，进而肿瘤进展，不仅使病情复杂化甚至危及生命。

EGFR-TKI所致ILD的主要临床症状常见以咳嗽（以干咳为主）起病，伴或不伴有渐进性加重的呼吸困难和发热^[90, 91]^。（1）咳嗽：82%-90%的患者有不同程度的干咳或有少量粘痰；（2）呼吸困难：多数患者表现为隐匿起病，渐进性，活动后呼吸困难；（3）发热：以低热更为常见，容易与肺部感染相混淆；（4）通常没有肺外表现，但可有一些伴随症状，如食欲减退、消瘦、乏力等；（5）最终可导致严重的双肺纤维化（蜂窝肺），引起呼吸衰竭、肺心病、心功能不全查体可闻及双下肺吸气末捻发音或湿啰音。肺功能检查为限制性通气功能障碍和弥散功能降低、可伴低氧血症^[92]^。胸部CT影像学表现多样、缺乏特异性，可有急性弥漫性间质性肺炎、闭塞性细支气管炎、隐源性机化性肺炎、嗜酸粒细胞性肺炎等的影像学改变特征，如双肺散在或融合的斑片状阴影、或弥漫性分布的磨玻璃样或网格状改变、小叶间隔增厚、多灶性肺实变、肺实变伴牵拉性细支气管扩张，最终可进展为肺纤维化、肺容积缩小、甚至蜂窝肺等^[93, 94]^，见图 2。

**2:**
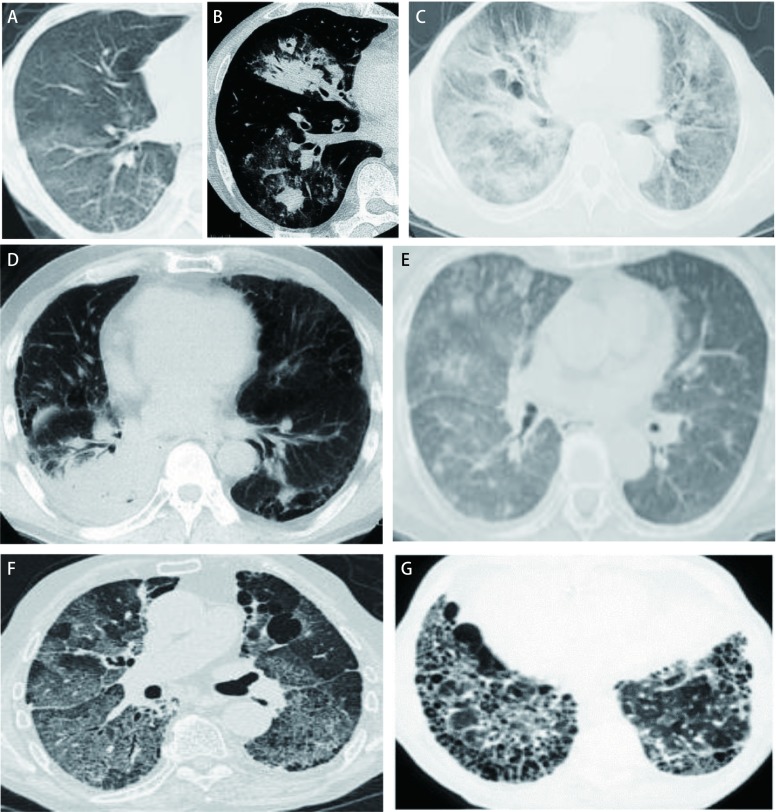
EGFR-TKI相关性ILD胸部CT特征。A：磨玻璃样改变；B：牵拉性细支气管扩张；C：急性间质性肺炎样改变；D：机化性肺炎样改变；E：嗜酸粒细胞性肺炎样改变；F：网织状改变；G：蜂窝状改变。 CT Features of EGFR-TKI-induced interstitial lung disease

### 4.4 EGFR-TKI相关性间质性肺疾病的诊断和鉴别诊断

#### 4.4.1 EGFR-TKI相关性间质性肺疾病的诊断

EGFR-TKI相关性ILD的诊断主要依据影像学诊断和排他性诊断。具体如下^[95]^：（1）近期使用过EGFR-TKI；（2）临床表现，影像学，病理学提示ILD；（3）除外其他引起ILD的病因；（4）停药后症状好转；（5）重新使用该药物，症状再发。

#### 4.4.2 EGFR-TKI相关性间质性肺疾病的鉴别诊断

服用EGFR-TKI的患者出现任何新发的呼吸系统症状都应谨慎评估，当胸部CT检查疑似存在药物相关性ILD的可能时，可考虑进行下列检查，排除以下情况（包括但不限于）：感染性疾病、肿瘤进展、肺栓塞或肺梗死、先前存在的间质性肺炎、放射性肺炎等。（1）血液学检查[血常规、C反应蛋白、降钙素原、病毒核酸检测、1, 3-β-D葡聚糖检测（1, 3-β-D-glucanassay test, G试验）、半乳甘露聚糖检测（Galactomannan test, GM试验）、隐球菌荚膜抗原、非典型病原体抗体、结核感染T细胞检测（tuberculous infection of T cells spot test, T-SPOT）、自身抗体等]；（2）痰涂片革兰氏染色和抗酸染色、痰培养（细菌、真菌和分枝杆菌）；（3）应在发热患者中进行血培养；（4）支气管镜检查（如可耐受）：采集支气管肺泡灌洗液（bronohoalveolarlavage, BAL）进行微生物学检测（与上述相同的病原体），同时经支气管镜肺活检（尽管肺组织活检并非必须，但可提高确诊率、缩短诊断时间、为治疗赢得时间。是否进行肺活检和活检方式的选择，取决于病灶部位、医疗机构技术条件和操作经验水平以及患者个体的危险因素）病理学检查帮助诊断。（5）动脉血气分析、肺功能（如可耐受）、心脏彩超、BNP等评估病情严重程度。

### 4.5 EGFR-TKI相关性间质性肺疾病的分级标准

目前临床尚无统一的EGFR-TKI相关性ILD的分级标准，本共识参考2017年NCI发布的CTCAE 5.0分级标准，药物相关性ILD分级标准如下^[22]^，见表 21。

**21:** ILD的分级标准 Grades criteria for ILD

分级	症状	活动能力	影像学改变	治疗干预
1	无症状；仅临床检查发现	正常	< 25%	无需干预
2	有症状	工具性日常生活活动受限	25%-50%	需要药物治疗
3	症状严重	个人日常生活自理活动^**^受限	51%-75%	需氧疗
4	危及生命的呼吸功能衰竭	卧床	> 75%	紧急抢救（如气管插管或气管切开）
5	死亡			
^*^工具性日常生活活动指做饭、购买杂物或衣服、使用电话、理财等。^**^日常生活自理活动实例包括沐浴、穿衣、吃饭、上厕所、服药等活动，即非卧床患者能够从事的活动。				

### 4.6 EGFR-TKI相关性间质性肺疾病的预防

使用EGFR-TKI前应对患者进行ILD危险因素评估；治疗期间加强对患者呼吸功能的监测和影像学检查，做到早发现、早停药、早治疗。

文献报道的预防方案包括使用EGFR-TKI前和使用EGFR-TKI时。具体预防方案详见表 22。

**22:** 使用EGFR-TKI前和使用EGFR-TKI时预防ILD的措施 Measures to prevent ILD before and during using EGFR-TKI

时间	预防措施
用药前	（1）应对患者进行ILD危险因素评估^[91]^； （2）如存在ILD危险因素和已有肺间质纤维化的患者，应谨慎使用EGFR-TKI^[94]^。
用药中	（1）避免与胸部放疗同步进行，可采用序贯治疗的方法^[91, 96]^； （2）避免与免疫检查点抑制剂同时使用； （3）加强对患者病情监测和随访，出现新发呼吸道症状或发热时，及时行胸部影像学检查。

### 4.7 EGFR-TKI相关性间质性肺疾病的处理（图 3）

**3:**
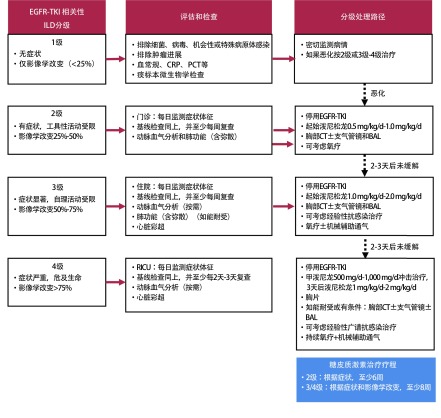
EGFR-TKI相关性ILD的分级处理 Management of ILD in patients taking EGFR-TKIs

ILD的治疗目标是抑制炎症反应，促进渗出吸收，防止肺间质纤维化，保护心肺功能。主要治疗方法包括氧疗、机械通气、糖皮质激素以及按需进行经验性抗感染治疗，密切监测病情变化，及时进行再评估和检查。

具体处理措施：（1）临床上一旦发生或怀疑ILD时，应立即停止EGFR-TKI；若有引起或加重ILD的合并用药（如博来霉素、胺碘酮等），可换用其他对ILD无影响的药物；（2）对于确诊或高度怀疑EGFR-TKI相关性ILD的患者，应立即开始以糖皮质激素治疗^[90]^，并注意补充钙及维生素D，监测血糖，预防消化道出血：①1级：密切监测症状体征和血液学检查，一旦恶化，按2级-4级治疗；②2级：起始泼尼松龙0.5 mg/kg/d-1.0 mg/kg/d或等效药物，持续2周-4周症状体征恢复后缓慢减量，总疗程至少6周^[90]^（1A）；③3级：起始泼尼松龙1.0 mg/kg/d-2.0 mg/kg/d或等效药物，持续2周-4周症状体征恢复后缓慢减量，总疗程至少8周^[91]^（1A）；④4级：甲泼尼龙500 mg/d-1, 000 mg/d冲击治疗，3天后泼尼松龙1 mg/kg/d-2 mg/kg/d，持续2周-4周症状体征恢复后缓慢减量，总疗程至少8周-10周^[91]^（1A）；（3）经验性抗生素抗感染治疗（按需或根据微生物学检查结果选择敏感抗感染药物）；（4）氧疗：推荐参照慢性阻塞性肺疾病氧疗指征，静息状态低氧血症[动脉血氧分压（partial pressure of artery oxygen, PaO_2_）≤55 mmHg，1 mmHg=0.133 kPa，或动脉血氧饱和度（oxygen saturation of arterial blood, SaO_2_）≤88%]的ILD患者接受长程氧疗，氧疗时间 > 15 h/d；（5）发生呼吸衰竭时行机械辅助通气。

### 4.8 EGFR-TKI相关性间质性肺疾病后再次用药问题

发生EGFR-TKI相关性ILD后，在肺部损害缓解前不宜再次使用EGFR-TKI。建议3级以上患者永久停药。1级-2级患者待肺间质损伤消退或治愈后，在全面充分评估临床获益与潜在风险后，且在没有其他种类全身系统治疗药物可以选择的情况下，方可考虑谨慎再次使用EGFR-TKI，治疗期间需密切观察病情，如果再次发生ILD，则永久停药。有一项研究报道了5例EGFR-TKI所致ILD恢复后的患者在再次使用EGFR-TKI的同时，口服泼尼松龙0.5 mg/kg，所有患者获得了部分应答。对于出现ILD的患者接受再次使用EGFR-TKI较停药患者有更长的整体存活时间（15.5个月 *vs* 3.5个月，*P*=0.029）^[97]^。

与EGFR-TKI所致皮肤和消化道不良反应相比，ILD发生率明显较低，但由于其病情发展迅速，及时停药后，病死率仍较高。因此，早期发现识别ILD以及提前采取相关的预防措施是至关重要的。从预防角度来讲，选择进行EGFR-TKI药物治疗的患者，应尽量避免一些高危患者，如高龄、男性，吸烟、既往有肺部放疗史等。对于服用EGFR-TKI药物的患者突发咳嗽加重、呼吸困难，应及时想到ILD，此时应及时监测肺部影像学变化而不是等待症状明显加重时再进行，应及时停药并给予恰当的处理以降低病死率。

## 5 小结

EGFR-TKI能改善晚期NSCLC患者的临床结局，而且多数EGFR-TKI所致不良反应是可防、可控的，停药后这些不良反应的级别可降低。患者宣教、及早识别和积极采取措施干预及治疗EGFR-TKI所致不良反应是关键。肿瘤科医生，包括其他多科室医生需共同努力和合作，采取综合防治措施如生活方式及药物干预等使EGFR-TKI所致不良反应最小化，避免不必要的减量或过早停药而中断有效治疗，从而提高患者治疗依从性和生活质量。

**参与本共识讨论和审定的专家（以姓氏汉语拼音为序）：**

胡洁（复旦大学附属中山医院呼吸科），侯炜（中国中医科学院广安门中医院肿瘤科），林丽珠（广州中医药大学第一附属医院肿瘤科），陆舜（上海交通大学附属上海市胸科医院肿瘤科），骆肖群（复旦大学附属华山医院皮肤科），茅益民（上海交通大学医学院附属仁济医院消化科），史美祺（江苏省肿瘤医院肿瘤科），王文梅（南京市口腔医院口腔粘膜病科），杨渤彦（中国医学科学院肿瘤医院综合科），于世英（华中科技大学同济医学院附属同济医院肿瘤科），姚煜（西安交通大学第一附属医院肿瘤科），周建英（浙江大学附属第一医院呼吸科），张燕群（北京大学肿瘤医院口腔科）

**执笔专家（以姓氏汉语拼音为序）：**胡洁、林丽珠、骆肖群、茅益民、周建英、张燕群
